# Changes of Gut Microbiota and Its Correlation With Short Chain Fatty Acids and Bioamine in Piglets at the Early Growth Stage

**DOI:** 10.3389/fvets.2020.617259

**Published:** 2021-01-05

**Authors:** Renli Qi, Xiaoyu Qiu, Lei Du, Jing Wang, Qi Wang, Jinxiu Huang, Zuohua Liu

**Affiliations:** ^1^Chongqing Academy of Animal Science, Chongqing, China; ^2^Key Laboratory of Pig Industry Sciences, Ministry of Agriculture, Chongqing, China; ^3^College of Animal Science and Technology, China Agricultural University, Beijing, China

**Keywords:** gut, gut microbiota, piglet, SCFA, bioamine

## Abstract

The change characteristics of intestinal microbial succession and the correlation with the production of two important types of bacterial metabolites (short chain fatty acids and bioamine) in piglets during the early stage were fully explored in this study. Six piglets from different litters with the same birth time were selected, weighted and euthanized at 1, 7, 14, 21, 28, 35, and 42 days of age. During this stage, the piglets grew quickly with gradual increases in blood levels of growth hormone and insulin, and in the intestinal developmental index and immunity. 16s rRNA analysis indicated the alpha diversity of colonic microbiome community was higher than ileum. However, the composition change in the ileal microbiota was more dramatic over time. *Lactobacillus* genus was the dominant bacteria in piglets' ileum while *Prevotella* and *Ruminococcaceae* genera were the dominant bacteria in colon up to weaning. Gut bacterial community of the piglets showed obvious differences between the three different phases: newborn, before weaning, and post weaning. This was similar to the morphological change pattern of pigs' gut. Total SCFA content in the colon of pigs showed almost a 20-fold increase at day 42 compared to the value at day 1. The percentage of acetic acid among the total SCFAs dropped quickly from 74.5% at day 1 to 36.5% at day 42, while butyric acid and propionic acid showed significant increases at the stage. The histamine level increased and putrescine level decreased markedly in the colon with time while the amounts of total bioamines, tyramine and spermidine were devoid of changes. Dozens bacteria taxa showed highly correlations with SCFAs and bioamines. These findings provide an expanded view of the dynamic pig gut and gut microbiome at the important early growth stage.

## Introduction

Newborn piglets have to adapt to the environment and acquire sufficient nutrition as soon as possible to ensure that they survive and grow. Although piglets grow fast during the lactation and nursery periods, their digestive system and immune organs are not fully developed, and their ability to protect against disease and stress is still poor. The separation from the sow and the change in food type could lead to increased stress stimulation to piglets at weaning (1, 2). Therefore, the physiological status of piglets changes quickly and considerably during the lactation stage and a few days after weaning (early nursery). Understanding and controlling the physiological changes in piglets during early life has important effects on improving pig health and growth performance.

Accumulating information about the gastrointestinal tract microorganisms of pigs has been obtained since the extensive application of “omics analysis” (3–7). It is believed that the gut of neonates is devoid of microbes prior to birth but rapidly undergoes a marked shift from an essentially germ-free state to harboring an extremely dense microbial population that eventually experiences microbial succession and establishes an adult-like microbial community (7–9). In addition to helping the host digest and absorb nutrients, gut microbes also affect and regulate the immune function, organ development, energy turnover, and disease formation of the host (10–13). A large number of diverse metabolites are produced by microbes in the digestive tract, including volatile short-chain fatty acids, bacteriocins, and amino acid derivatives (14–16). Furthermore, gut bacteria also affected the production, transformation, and absorption of the plant-derived secondary metabolites in animals. Many of the bacterial secondary metabolites are related to the development and physiological homeostasis in animals closely. For instance, urolithin A, a bacterial metabolites produced from the metabolism process of plant-derived polyphenols in gut (17), and it has been proved could exerts anti-obesity effects in animals (18). These beneficial or harmful bacterial primary and secondary metabolites enter the circulatory system and then trigger or affect the functions of other organs and tissues (e.g., brain) via complex signal cascades.

Gut microbe colonization and succession in the early postnatal period are well-known to facilitate immune maturation and will have long-term important effects on the healthy growth and development of animals throughout their lives. Many sundry environmental factors or the stress caused by physiological changes affect the colonization and composition of the intestinal bacterial community in frail baby pigs at the early stage, including the living environment, lactation, nursing, feeding, weaning, and contact with the mother pig and stockman (19–21).

We have fully understood the importance of the intestinal microbiota for early growth in pigs, but to date, most studies have been based on the fecal microbiome. The major aim of this study was to explore the change traits of the gut morphology, intestinal microbiota and gut bacterial metabolites in infant pigs from the newborn stage to post-weaning based on continuous analysis of intestinal samples to reveal the close relationship between the gut bacteria and major bacterial functional metabolites in pigs at the early growth stage. We showed that the rapid development characteristics of the gut and gut microbiota of pigs in early life. The amount and structure of short-chain fatty acids and biogenic amines in the piglets' gut are closely related to intestinal microbiota composition. Creep feeding and weaning had important implications for gut development and gut microbial succession in swine. The results from the present study will provide assistance for early health maintenance for pig production.

## Materials and Methods

### Ethics Statement

All animal experiments were conducted pursuant to the Regulations for the Administration of Affairs Concerning Experimental Animals (Ministry of Science and Technology, Beijing, China, revised June 2014). The Ethics Committee of the Chongqing Academy of Animal Science approved the animal experimental protocols (CAAS-S2018-11, Chongqing).

### Animals and Collection of Samples

The animal experiments were performed at the Laboratory Animal Center of the Chongqing Academy of Animal Science from April to July 2018. The Rongchang pig, a famous local meat-type pig breed in China, was used as the animal model in this study. Twenty purebred Rongchang sows (second parity) with a similar expected delivery date were selected for this study. Each sow was individually housed in a different environmentally controlled room under standard management with access to commercial feed and clean water. The newborn piglets were co-housed with their mother pigs after delivery. Suckling piglets were offered a common creep feed *ad libitum* at day 7 and weaned at day 28. All piglets remained in nursing pens for other 2 weeks until day 42. During this stage, the piglets were fed with commercial solid feed and had free access to clean water post weaning, while sows were removed from the piglets at day 28. All piglets were weighed at each time point for the sample collection, and the occurrence of diarrhea was assessed two times a day (monitoring times: 10 a.m. and 4 p.m.). Six piglets, half males and half females, were randomly selected from different litters and then sacrificed immediately by jugular puncture after anesthesia with Zoletil 50® (Virbac, Carros, France) at days 1, 7, 14, 21, 28, 35, and 42, respectively. Their venous blood, intestinal samples, and gut digesta were collected. Digesta samples were collected from the middle ileum and middle colon sections, placed into sterile polypropylene centrifuge tubes, snap frozen in liquid nitrogen and kept frozen at −80°C until DNA extraction. For intestinal morphological analysis, the mid-ileum (~10 cm from the ileal-cecal junction) segments were sampled and fixed in buffered formalin (10%) at 4°C for morphometric analysis. The adjacent ileum was also fixed overnight in a 2.5 % glutaraldehyde solution at 4°C, and then, these samples were treated for observation by electron microscopy. The mucosa samples from the colon were harvested by scraping with a sterile glass microscope slide, rapidly frozen in liquid nitrogen and stored at −80°C for sIgA content analysis.

### 16S rDNA Amplicon Sequencing

Microbial genomic DNA was extracted from intestinal digesta samples using a QIAamp DNA Stool mini kit (Qiagen, GmbH Hilden, Germany). DNA was quantified with a NanoDrop 2000 spectrophotometer (Thermo Fisher, DE, USA), and the integrity was checked by agarose gel electrophoresis. PCR primers flanking the V3–V4 hyper variable region of bacterial 16S rDNA were designed. The forward primer was 341F (5′-CCTAYGGGRBGCASCAG-3′), and the reverse primer was 806R (5′-GGACTACNNGGGTATCTAAT-3′). The PCR resulting amplicons were gel purified and sequenced on the Illumina MiSeq platform. The sequences were clustered into OTUs (Operational Taxonomic Units) with 97% consistency, and a representative sequence of OTUs was selected. Sequences for each OTU were picked and aligned using QIIME version 1.9.1 and GreenGenes version 13_8 as the reference database. Taxon-dependent analysis was conducted using the Ribosomal Database Project (RDP, version 2.2) classifier. To identify the genomic features of taxa differing in abundance between classes, the LEfSe (Linear Discriminant Analysis Effect Size) algorithm was used with the online interface Galaxy (http://huttenhower.sph.harvard.edu/lefse/).

### Examination of Intestinal Morphology

The ileum and colon samples were embedded with paraffin wax and sectioned at 5 mm on a sliding microtome. Then, the sample sections were stained with hematoxylin and eosin (HE, Solarbio, Beijing, China). Villus height and crypt depth as measures of the ileum and mucosal layer thickness of the colon were evaluated under a light microscope using a 1/100 ocular scale (Olympus, Japan). The ileum and colon sections were also stained with the Periodic Acid-Schiff stain (PAS, Solarbio) to observe the morphology and distribution of goblet cells in the intestinal epithelium. Five complete intestinal villi were selected from each sample slice, and the number of goblet cells per 100 intestinal epithelial cells was counted.

### Western Blotting Analysis

The protein expressions of Occludin and β defensin in the colon wall were determined by the standard Western blot method with the GAPDH protein as a loading control. The anti-Occludin (#13409-1-AP) and anti-GAPDH (10494-1-AP) primary antibodies were obtained from Proteintech (Wuhan, Hubei, China). The anti-β defensin (#bs-1296R) primary antibody was obtained from Bioss (Bioss Biotech, Beijing, China). The horseradish peroxidase-conjugated secondary antibody was obtained from Cell Signaling Technology Co.

### qRT-PCR

The mRNA levels of Toll-like receptors 2 and 4 gene were detected by the qRT-PCR method. Briefly, total RNA was extracted from clean ileum samples by using an RNAplus kit (TaKaRa, Dalian, China). Total RNA was reverse-transcribed to cDNA using a PrimeScript RT Reagent Kit (TaKaRa) according to the manufacturer's instructions. qPCR was performed using the Q6 qPCR system with SYBR Premix Ex Taq II (TaKaRa) and normalized using the beta actin gene as the endogenous control. All reactions were set to 3 replicates, and the expression levels of genes were expressed as fold-change using the 2^−ΔΔCT^ method.

### Biochemical Analysis

The concentrations of growth hormone, insulin, Immunoglobulin G (IgG), and IgM in the blood samples were evaluated using commercial enzyme-linked immune sorbent assay (ELISA) kits (mlbio Co., Ltd., Shanghai, China).

### Short-Chain Fatty Acid Concentrations

The content of SCFAs in the colonic digesta was determined by using an ISQ Lt GC-MS (Thermo Fisher, USA) equipped with a flame ionization detector. A TG WAX column (30 m × 0.25 mm × 0.25 μm, Thermo Fisher) was used to separate the SCFAs. A sample injection volume of 5 μL was automatically injected into the inlet, which was kept at 240°C with a 75:1 split ratio. The carrier gas was helium at a flow rate of 1.0 mL/min. The temperatures of the flame ionization detector (FID, Thermo Fisher) and injector were 250 and 200°C, respectively.

### Biogenic Amines Concentrations

The content of biogenic amine in the colonic digesta was detected with a U3000-HPLC system (Thermo Fisher, USA). Added the digesta samples (0.75 g) into 5 mL of 5 % perchloric acid and then ultrasonic extracted for 30 min. Mixed 1 ml extraction solution, 0.2 ml NaOH (2 M) and 100 ul benzoyl chloride, and then incubated at 40°C for 30 min, then added methanol in the mixture to stop the reaction. The mixture was analyzed by the HPLC system after filtration. The chromatographic conditions: UV detection wavelength 254 nm, column temperature 35°C, mobile phase A was 90% acetonitrile/10% 0.01 mol/L ammonium acetate solution (containing 0.1% acetic acid), and mobile phase B was 10% acetonitrile/90% 0.01 mol/L ammonium acetate solution (containing 0.1% acetic acid), the flow rate was 0.8 mL/min, Column: Syncronis C18 (250 mm × 4.6 mm × 5 μm).

### Statistical Analysis

Statistical analysis was performed using the SPSS 22.0 (SPSS, Chicago, IL, USA) and the R statistical package version 3.5.1. The mean values were examined using one-way ANOVA followed by Tukey–Kramer test. The level of significance was set at *P* < 0.05. The data were expressed as mean ± standard error (S. E.). The diversity and richness indexes (Chao index, Shannon index), the phyla and genus relative abundance were compared between ages using Kruskal–Wallis *H* tests. The similarities between samples at different times were analyzed by a non-metric distance scaling (NMDS) method based on the Bray–Curtis distance matrix. The Sankey analysis is used to visualize the proportion change of major bacteria at different time.

## Results

### Early Growth and Development of Piglets

The newborn Rongchang piglets from 20 different litters with similar birth dates were selected and used in this study. Table 1 shows the early growth states and blood indexes of the piglets from days 1–42. The body weight (BW) of the piglets increased rapidly from 0.836 kg at 1 day of age to 8.425 kg at 42 days (*P* < 0.0001). The levels of growth hormone (GH) and insulin in the blood of the piglets also increased successively (*P* < 0.0001). GH levels did not vary before 14 days of age, increased quickly from 21 days of age (more than 2.5-fold that on of newborn piglets), and increased significantly again after 35 days of age (more than 4.0-fold that of newborn piglets). The blood level of insulin showed a gradual increase with the growth of piglets. The blood concentrations of immunoglobulins IgG and IgM clearly declined before 14 days of age and then exhibited an increasing trend with time. Interestingly, the concentrations of blood lipids, triglycerides and total cholesterol in the blood of the pigs showed an overall reduction trend at this stage. This may be caused by the active metabolism and substantial consumption of energy with the fast growth of piglets at this stage. In addition, the frequency of diarrhea in the piglets increased from 10 days of age at this stage, especially within a few days after the piglets were fed creep feed and on other days after weaning. The incidence of diarrhea decreased significantly after 35 days of age (*P* < 0.001).

**Table 1:** Early growth and blood indexes of Rongchang piglets.

**Index**	**D1**	**D7**	**D14**	**D21**	**D28**	**D35**	**D42**	**ANOVA *P*-value**
Body weight, kg	0.84 ± 0.08f	1.53 ± 0.21e	3.25 ± 0.30d	3.94 ± 0.11d	5.53 ± 0.25c	6.73 ± 0.28b	8.63 ± 0.35a	*P* < 0.0001
Serum growth hormone, ng/mL	14.07 ± 0.88c	10.07 ± 0.36c	12.68 ± 0.81c	36.63 ± 9.27b	38.55 ± 3.61b	64.72 ± 7.74a	68.25 ± 11.62a	*P* = 0.0002
Serum insulin, pg/mL	11.13 ± 1.40e	12.37 ± 0.72e	14.52 ± 1.44d	17.61 ± 1.60c	21.11 ± 0.84b	30.09 ± 1.73a	32.25 ± 0.89a	*P* < 0.0001
Serum triglyceride, mmol/L	0.66 ± 0.13b	0.89 ± 0.08a	0.65 ± 0.19b	0.64 ± 0.10b	0.52 ± 0.16b	0.38 ± 0.06c	0.22 ± 0.03d	*P* = 0.0145
Serum total cholesterol, mmol/L	1.24 ± 0.10d	2.77 ± 0.38b	3.70 ± 0.47a	2.99 ± 0.21b	1.96 ± 0.39c	1.47 ± 0.14d	1.36 ± 0.16d	*P* < 0.0001
Serum immunoglobulin G (IgG), mg/mL	4.06 ± 0.35b	3.63 ± 0.12c	3.48 ± 0.09d	3.70 ± 0.21c	3.78 ±0.22c	4.28 ± 0.33b	4.62 ± 0.29a	*P* = 0.0342
Serum immunoglobulin M (IgM), mg/mL	0.13 ± 0.04b	0.06 ± 0.02e	0.05 ± 0.02e	0.09 ± 0.02d	0.11 ± 0.03c	0.20 ± 0.06a	0.24 ± 0.03a	*P* = 0.0027
Diarrhea, %	3.7%d	8.9%c	23.5%a	20.2%a	16.4%b	5.1%d	4.6%d	*P* < 0.0001

*N = 6, the data are presented as the mean ± S.E., values within a row with different superscripts differ significantly at P < 0.05 (ANOVA)*.

### Gut Development and Immune Functions

**Figure 1:**
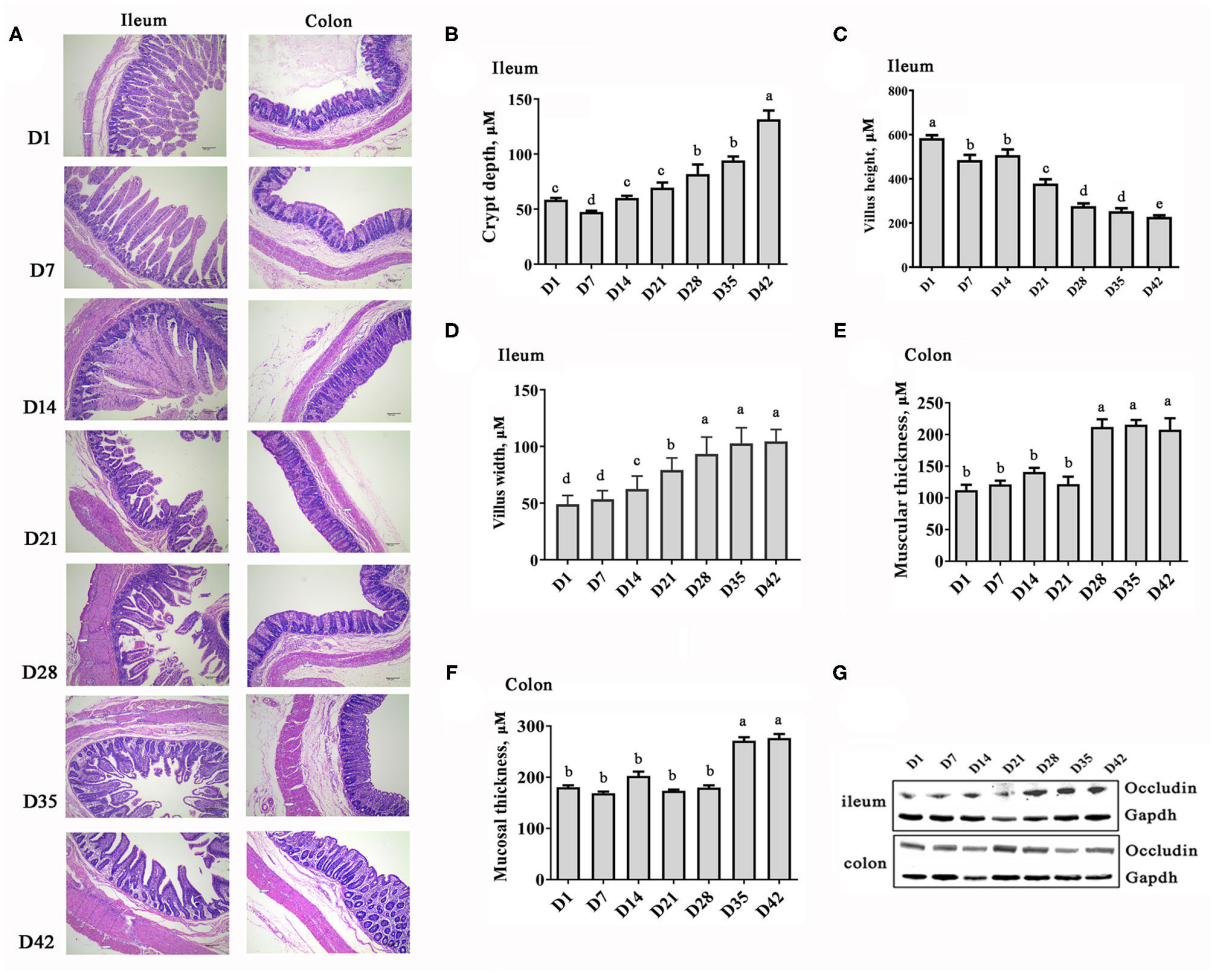
Changes in the intestinal morphology of piglets at the early growth stage. **(A)** HE staining images of ileum and colon sections (40× magnification). **(B)** Depth of ilealcrypt. **(C)** Height of ileal villi. **(D)** Width of ileal villi. **(E)** Thickness of colonic muscular layer. **(F)** Thickness of colonic mucosal layer. **(G)** Protein abundance of Occludin. *n* = 6, the data are presented as the mean ± S.E., different letters on the bars indicate significant differences at *P* < 0.05 (ANOVA).

Figure 1 illustrates the intestinal development and morphological changes in the Rongchang piglets from 1 to 42 days of age. Decreases in the villus height and significant increases in villus width as well as crypt depth in the ileum were observed with time (Figures 1A–E). The colonic muscular layer and mucosal layer thickness increased after weaning (*P* < 0.001, Figure 1F). In addition, the protein abundance of Occludin, a key tight-junction protein, was increased to varying degrees in the ileum and colon of the piglets over time (Figure 1G). These results indicate that the intestinal structure becomes stable and matures quickly with the growth of pigs.

**Figure 2:**
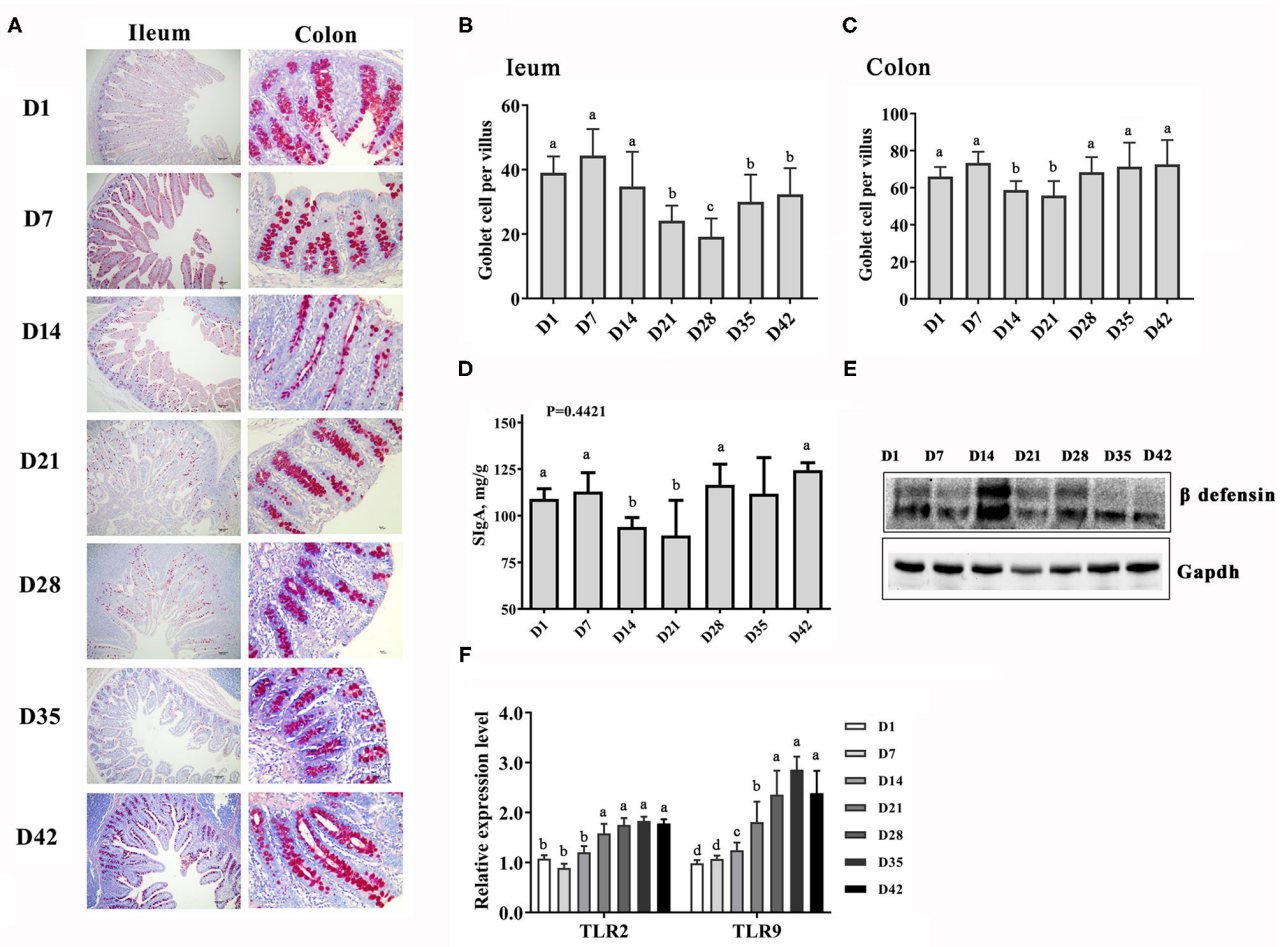
Changes in the intestinal immune function piglets at the early growth stage. **(A)** PAS staining of goblet cells in the gut sections, 40× magnification. **(B,C)** Counts of the goblet cells in the ileum and colon. **(D)** Content of secretory IgA in the intestinal mucosa. **(E)** Protein abundance of β defensin. **(F)** Expression levels of the TLR2 and TLR9 genes in the ileum. *n* = 6, the data are presented as the mean ± S.E., different letters on the bars indicate significant differences at *P* < 0.05 (ANOVA).

The intestinal barrier and immune function were also strengthened quickly with the growth of the piglets, as evidenced by the increase in the number of immune cells and in the level of mucosal immune factors (Figure 2). However, it should be noted that gut morphology and function were affected even when damaged by frequent diarrhea in some piglets between days 14 and 28. The number of goblet cells in the ileum and colon of piglets decreased mainly between days 14 and 28 and then increased, and the cells became larger over time (Figures 2A–C). Similarly, the level of secretory IgA (SIgA) in the colonic mucosa of piglets also decreased at days 14–21 and then rebounded (Figure 2D). The beta defensin protein abundance was higher at days 14–28 than at other time points (Figure 2E). Toll-like receptors (TLRs), a superfamily of pattern-recognition receptors, play a pivotal role in host innate immunity against pathogen infection (22). The TLR2 and TLR9 genes in the ileum showed increased expression of mRNA from 21 days (Figure 2F). These results indicated that a secure intestinal immunity system was formed in swine after weaning.

### Changes of Intestinal Microbiota

**Figure 3:**
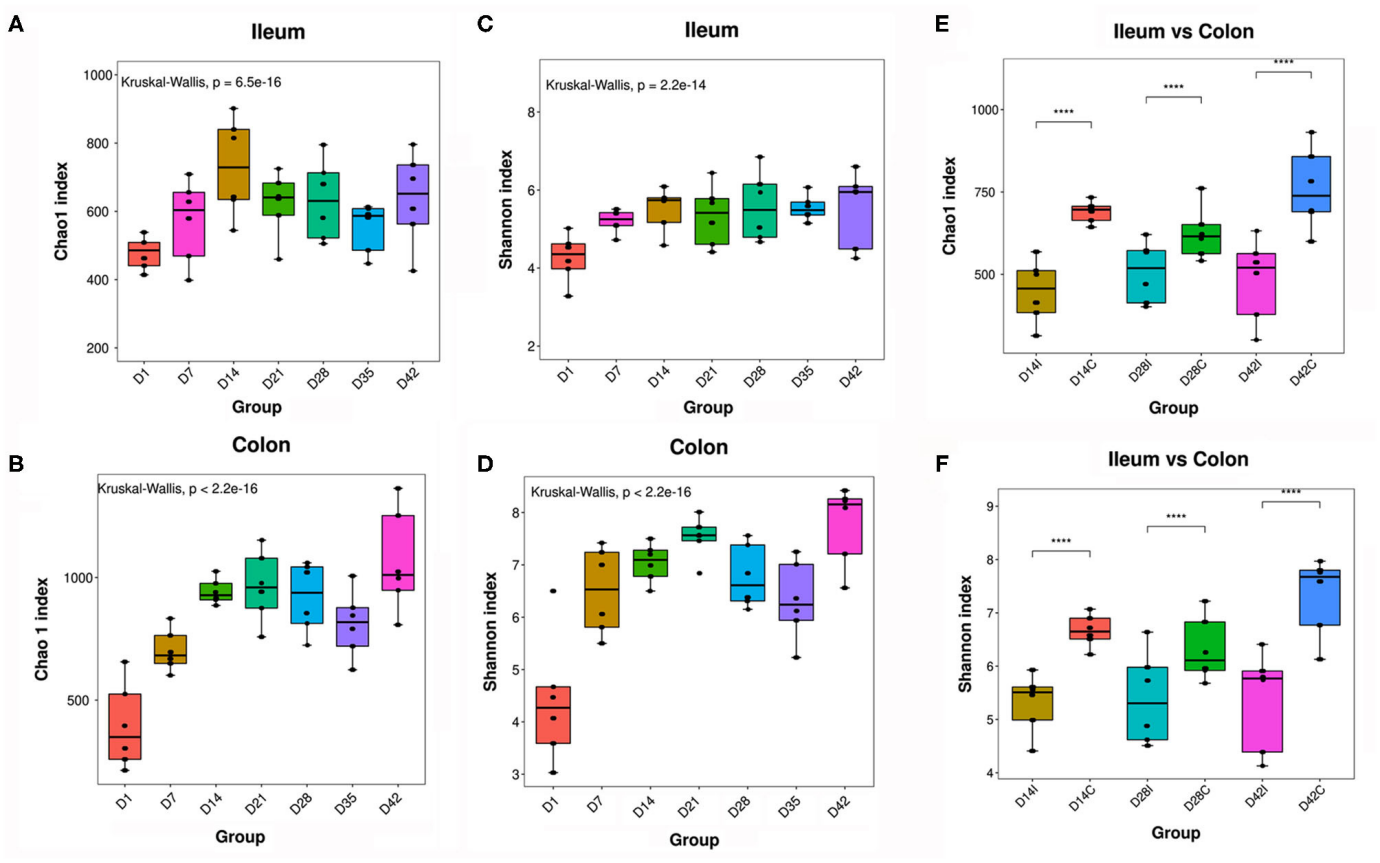
Alpha diversity of piglets' intestinal microbiota. **(A,B)** Chao1 index of the ileal and colonic microbiota. **(C,D)** Shannon index of the ileal and colonic microbiota. **(E,F)** Comparison of alpha diversity between the ileal and colonic microbiota, *n* = 6, *P* < 0.05 was considered statistically significant (Wilcoxon Kruskal–Wallis test). ****means *P* < 0.0001.

The ileal and colonic microbiomes of the piglets were analyzed during the early growth stages by 16S rDNA sequencing. In the gut of the piglets, the Chao1 index (for microbial community richness) and the Shannon index (for microbial community diversity) showed significant increases with time, specifically in the first week (Figures 3A–D). In addition, the alpha diversity of the colonic microbiota was clearly higher than that in the ileum at the same time point (Figures 3E,F).

**Figure 4:**
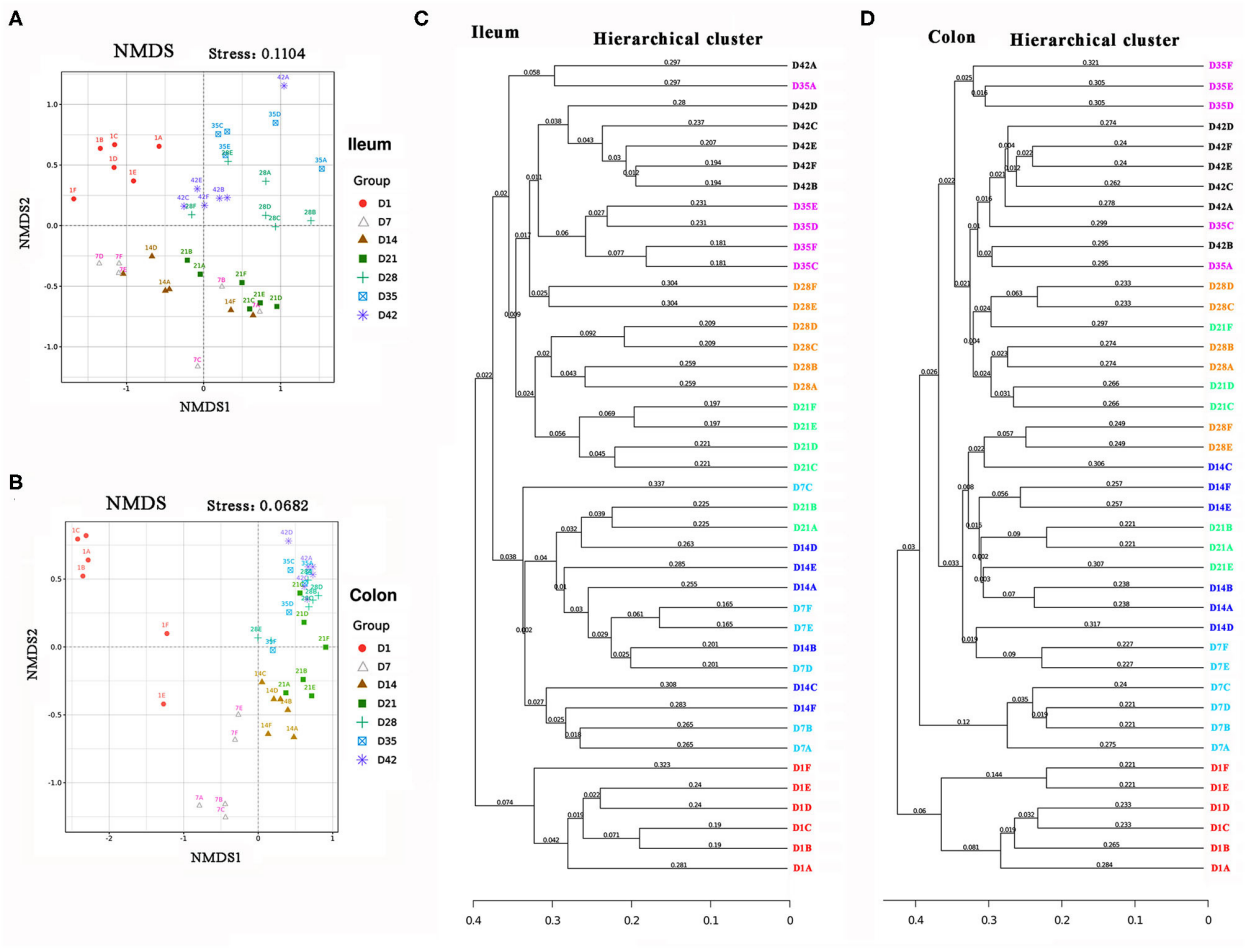
Beta diversity of piglets' intestinal microbiota. **(A,B)** Unweight non-metric multidimensional scaling (NMDS) analysis of the ileal and colonic microbiota. **(C,D)** The hierarchical cluster of the intestinal microbiota.

The gut microbial communities at different time points were then grouped by hierarchical clustering and ordinated by unweight unifrac nonmetric multidimensional scaling (NMDS). The hierarchical clustering and NMDS analysis indicated that the gut microbial samples from the piglets formed three separate clusters by time point: day 1, days 7–21, and 28–42 (Figures 4A,B). In addition, results from the hierarchical clustering analysis about gut microbiota composition were similarly (Figures 4C,D). This time-phased change in the gut microbiota was similar to the developmental traits of the physiological states and intestinal morphology of pigs.

**Figure 5:**
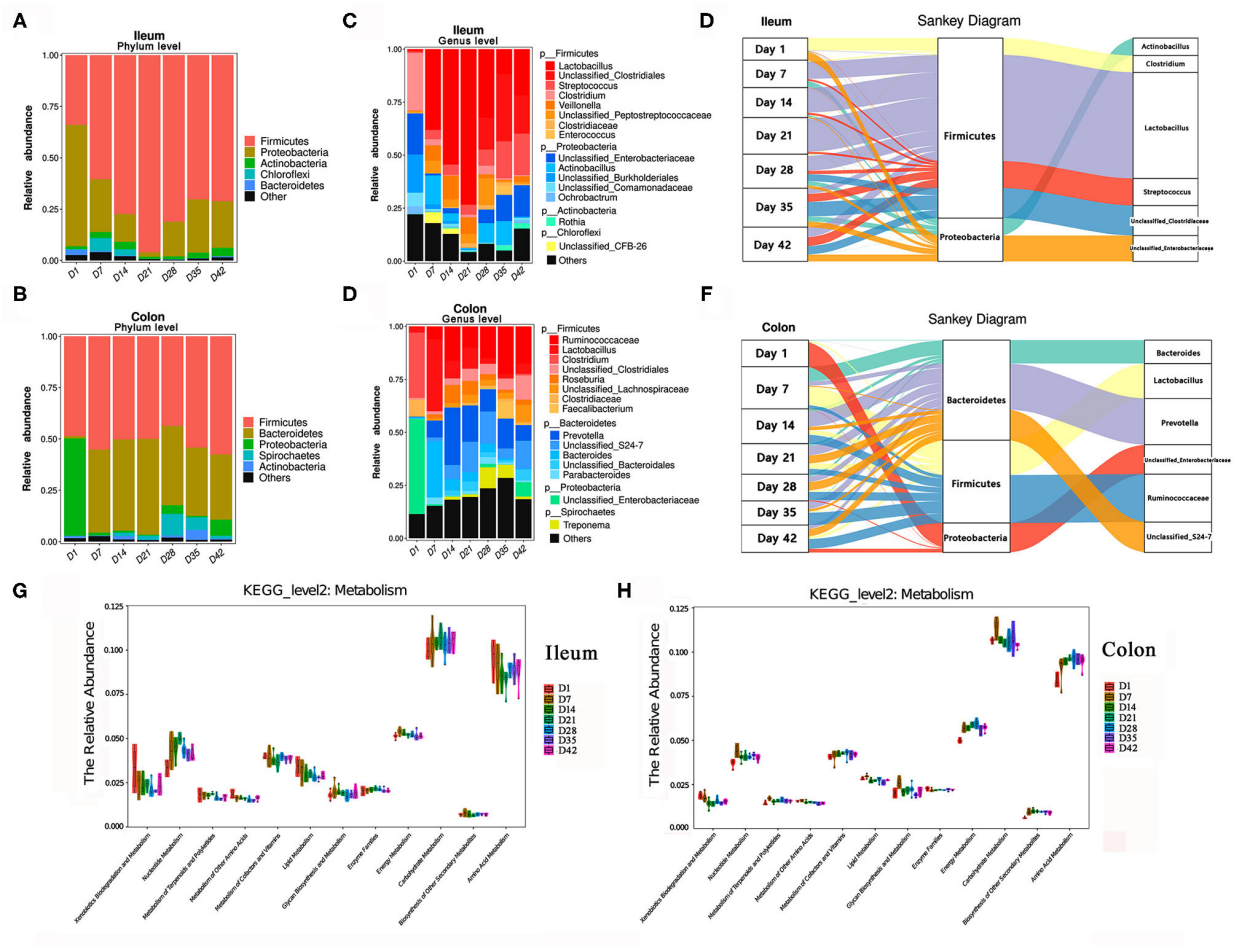
Changes in the composition of the intestinal microbiota in piglets. **(A,B)** Change in the relative abundance of the top bacterial phyla in piglets. **(C,D)** Change in the relative abundance of the top bacterial genera in piglets. **(E,F)** Sankey analysis of the change of ileal and colonic microbiota. **(G,H)** Changes in the relative abundance of known KEGG pathways (metabolism, level 2) for ileal microbiota and colonic microbiota. The functional contributions of the gut microbiota were assessed using the PICRUSt tool.

In addition to the changes over time, the microbiota compositions in the proximal intestine (ileum) and distal intestine (colon) were clearly different. As shown in Figures 5A,B, the most abundant phyla displayed distinct profiles in the ileum and colon. *Firmicutes, Proteobacteria*, and *Actinobacteria* were the top three bacterial phyla in the ileum, while *Firmicutes, Bacteroidetes*, and *Proteobacteria* were the top three phyla in the colon during the early growth stage. Among these bacteria, the relative abundance of the *Firmicutes* phylum showed a tendency to increase with the growth of baby pigs in the ileum but showed a higher stability in the colon than in the ileum. The bacterial composition in the ileum of the piglets showed drastic changes before day 21, which was characterized by a marked increase in the relative abundance of the *Firmicutes* phylum (from 33.98% at day 1 to 95.01% at day 21) and a rapid decrease in the abundance of *Proteobacteria*. After 21 days, the relative abundance of *Firmicutes* decreased to ~71%, the abundance of *Proteobacteria* increased to ~22–25%, and the bacterial composition became more stable. In the colon of piglets, the relative abundance of the *Firmicutes* phylum, which was the first bacterial phylum, was consistently maintained at more than 50%. *Bacteroidetes* replaced *Proteobacteria* as the second most abundant phylum quickly after the birth of the pigs.

As shown in Figures 5C,D, at the genus level, the top three dominant bacterial genera in the ileal digesta were *Lactobacillus, Streptococcus, Clostridium*. In the colonic digesta, the top three bacterial genera were *Prevotella, Ruminococcaceae*, and *Lactobacillus. Lactobacillus* genus had the greatest numerical relative abundance in the ileum of pigs during lactation, especially before weaning followed a significant decrease in the abundances of *Lactobacillus* in both the ileum and colon after weaning, and the abundance then rebounded a few days later. When the piglets were exposed to solid feed after 7 days, the relative abundance of *Prevotella* and *Ruminococcaceae* genera in the colon began to increase significantly, which was useful for food digestion and nutrient intake (especially carbohydrates) (23, 24). Figures 5E,F illustrates the dynamic changes in dominant bacteria in the piglets during early life period. Additionally, the potential metabolism functions of the microorganisms in piglets were predicted by using the PICRUSt tool (Figures 5G,H). Clearly, amino acid metabolism, carbohydrate metabolism, and energy metabolism were the top three metabolisms with highly abundances in the little pigs. Energy metabolism and carbohydrate metabolism showed relative stable with the growth of piglets. Relative abundance of amino acids metabolism showed decrease in ileum but increase in the colon with the growth of piglets.

**Figure 6:**
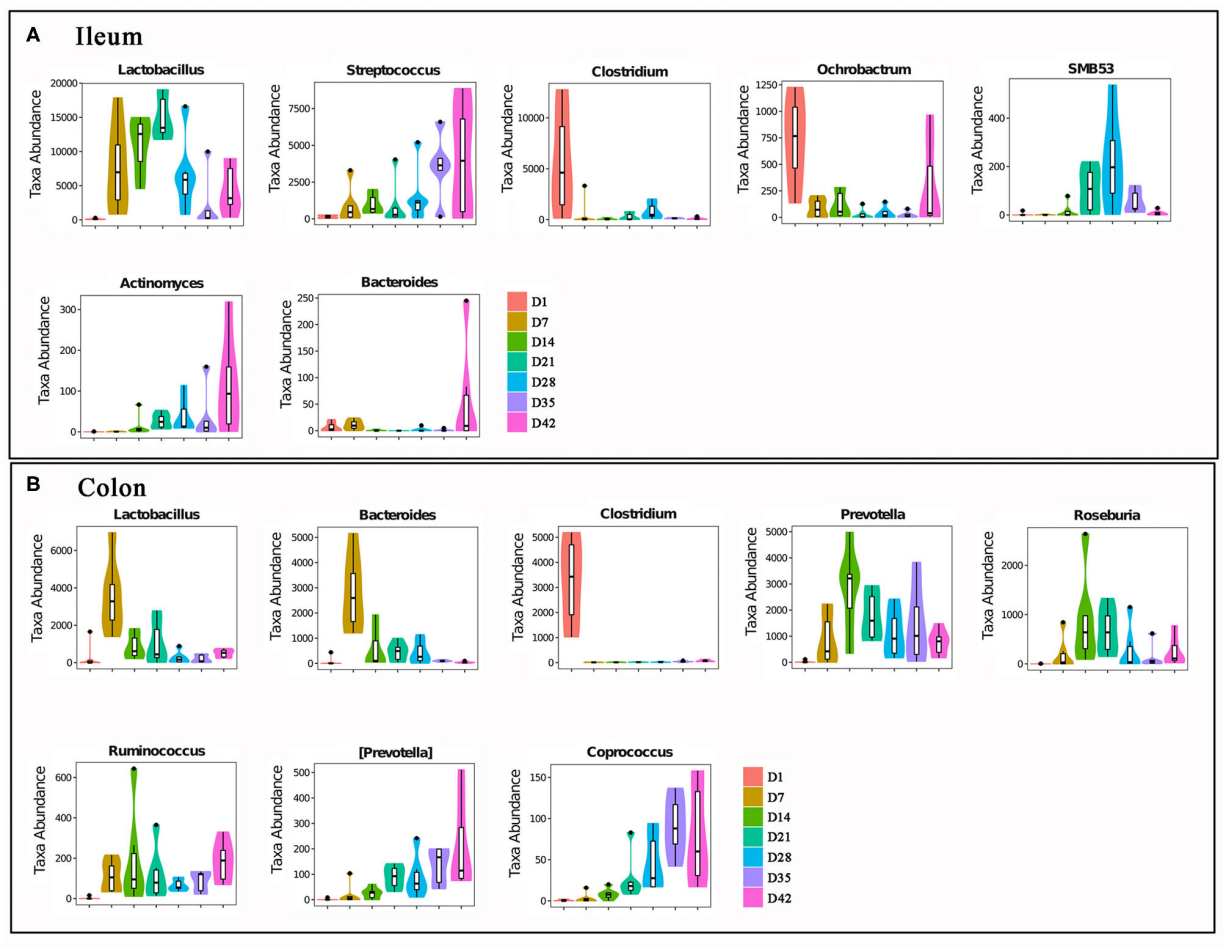
The major intestinal bacteria with significant abundances changes. **(A)** Ileal bacteria genera. **(B)** Colonic bacteria genera. All bacteria in the figure were changed in abundance significantly. The differences were analyzed by Metastats method, *n* = 6, *P* < 0.05.

Figure 6 shows changes in the abundances of major abundant bacterial genera with statistically significant differences (*P* < 0.05) that determined by the Metastats analysis method. Similarly, the changes in composition of the gut bacterial community also showed obvious differences between the three phases: day 1 (newborn), days 7–21 (before weaning), and days 28–42 (post-weaning).

### SCFA and Bioamine

**Figure 7:**
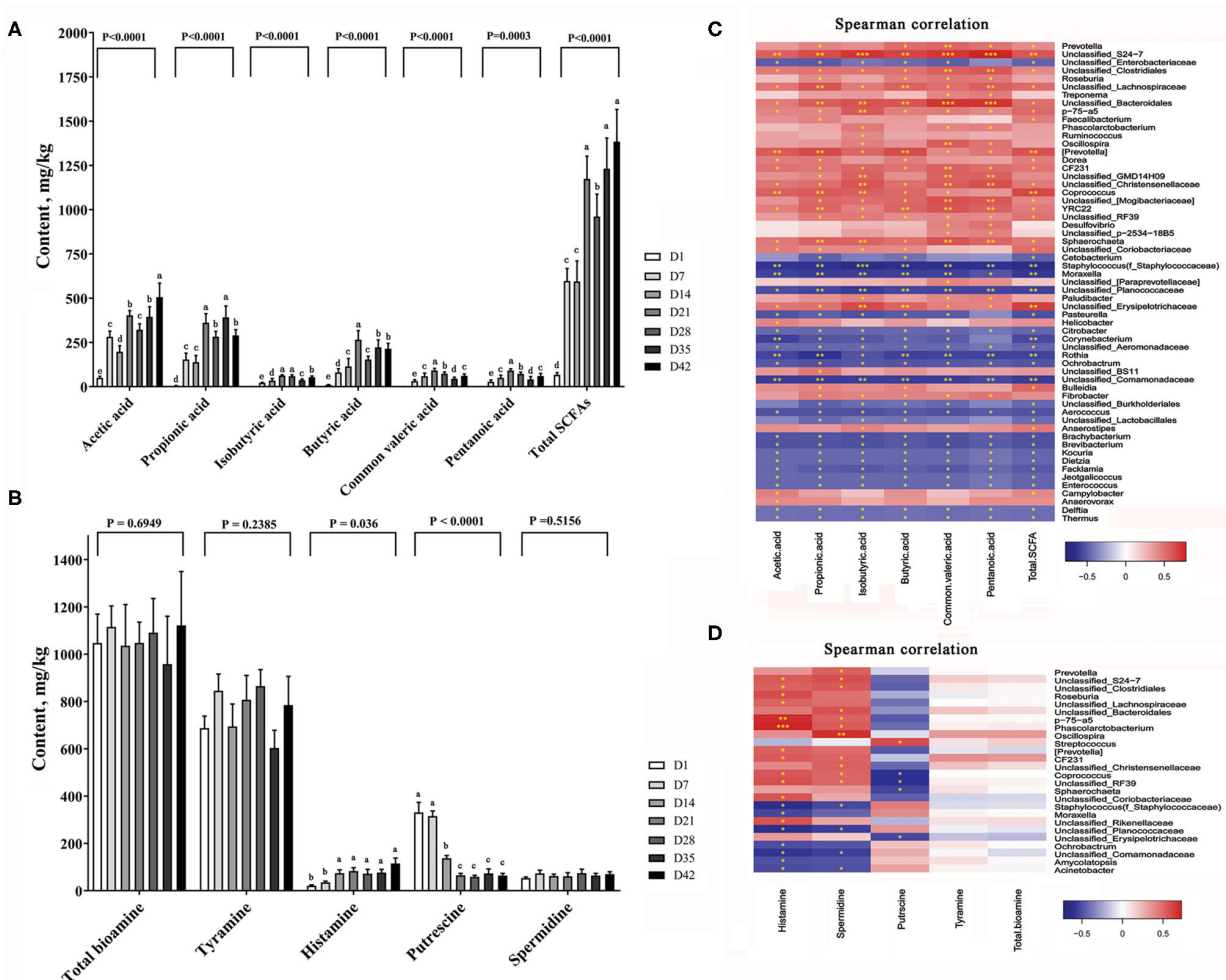
Changes of major bacterial metabolites. **(A)** SCFA content in the colon of piglets, **(B)** Bioamine content in the colon of piglets. The data are presented as the mean ± S.E., *n* = 5, and different letters on the bars indicate significant differences at *P* < 0.05 (ANOVA). **(C)** Spearman's correlations between intestinal microbiota and SCFA; **(D)** Spearman's correlations between intestinal microbiota and bioamine. The colors range from blue (negative correlations) to red (positive correlations). Significant correlations are noted by ***P < 0.001, **P < 0.01, and *P < 0.05.

Bacterial metabolites with functional activity (known as post-biotics) are the main mediators of the physiological functions of gut microbes (16). We next analyzed the contents of short-chain fatty acids (SCFAs) and bioamines, two major classes of bacterial metabolites, in the colonic digesta of piglets. The amount of total SCFAs in the gut increased markedly with time (Figure 7A). At day 7, the amount of total SCFAs in the colon contents of piglets was almost 9-fold that at day 1. When the piglets grew to 42 days of age, the amount of total SCFAs in the pigs was almost 20-fold that in pigs at 1 day of age. The SCFA composition also varied greatly in early life. The percentage of acetic acid in the total SCFAs dropped from more than 74.5% on day 1 to 36.5% on day 42 (*P* < 0.0001). Both butyric acid and propionic acid showed significant increases in percentage during this stage.

Biogenic amines (bioamines) are a class of nitrogen-containing organic active substances that function in neuromodulation in animals (25). Figure 7B shows the changes in total bioamines and four major bioamines in the colon contents of piglets with time. The amount of total bioamines showed a slight increase in the colonic digesta between days 1 and 42 (*P* = 0.6949). Tyramine and spermidine were relatively stable and showed no significant changes in content at this stage (*P* = 0.2385 and 0.5156). However, the histamine content increased markedly with time (*P* < 0.001), while the putrescine content was reduced by ~65% after 14 days (*P* = 0.036).

By the Spearman correlation analysis we established a link between the colonic metabolites content and the abundance of intestinal bacteria. Figure 7C shows the correlation between SCFAs and major bacteria taxa. Dozens of microbe showed highly positive or negative correlation with the SCFA contents. Figure 7D shows the correlation between the different bioamines and gut bacteria. We found *P-75-a5, phascolarctobacterium, oscillospira* genera were strongly positive correlated with most of the bioamines contents.

## Discussion

There is no doubt that insight into the dynamic traits of the development and function of the digestive tract in pigs and following the administration of nutrition or drug intervention is extremely important for pig performance. In addition, the intestinal structure and functions in pigs are very similar to those in humans, and a study based on swine will also be important for studies related to the human intestine (26, 27).

Our present work systematically explored the physiological changes in Rongchang piglets in early life between 1 and 42 days of age (lactation and early nursery), especially changes in intestinal morphology, intestinal microbe colonization, and major bacterial metabolites. The infant pigs showed rapid growth of physiological indexes (BW, growth hormone, and other blood biochemical traits) under normal feeding conditions. The fast growth and development was also observed in the pigs' digestive tract, which manifested as increases in intestinal mucosa thickness and immunity factors. Microbes colonized pigs' gut after the born immediately, with significant elevation of diversity and richness mainly presented in the 1st week of age, which was followed by turbulence for 21 days, and then, a stable microbial community was formed after weaning (day 28). Additionally, the overall amount of bacterial metabolites in the gut also increased rapidly, and the composition was dynamic, which may be caused by the change in the gut microbiota structure. It can be deduced that these changes may affect the development and function of the host gut.

Many previous studies have identified the core microbiota from the gut microbiome community at different pig developmental stages through metagenomic and metatranscriptomic analyses (5, 7, 19, 21). A number of gut bacteria have been identified for their beneficial or harmful physiological functions. Colonization and changes in beneficial functional microbes (e.g., *Lactobacillus* and *Prevotella*) have great potential to contribute to feed intake, feeding efficiency, obesity, and muscle growth of pigs at a specific time (24, 28, 29). Conversely, the invasion by harmful bacteria (e.g., *Erysipelotrichaceae*) is threats to host health (30). To further improve feeding efficiency and effective nutrient utilization, some studies have suggested that individualized breeding and feeding programs should be developed in the pig industry based on the pigs' different intestinal microbiota compositions and enterotypes (5, 31, 32).

Recently, a longitudinal analysis investigated the evolution of pigs' fecal microbiota composition from post-weaning to finishing. The results showed that the pigs were classified as two different enterotypes dominated by either *Lactobacillus* or *Prevotella* at day 52 (5). Similarly, we also confirmed that the dominant bacteria in the ileum and colon in piglets were *Lactobacillus, Prevotella* and *Ruminococcaceae* respectively, during early life. The abundance change trends in the gut bacteria in the present study were consistent with those in a previous study conducted in pigs. The top bacterial genera play important roles in the control of the intestinal microenvironment and nutrient absorption. Similar to previous report (3, 33), our results showed again that there are clear and distinct differences in the composition of the pig intestinal microbiome moving from the proximal end of the intestinal tract to the distal end. The colon bacterial community diversity was higher than that in the ileum at the same time. However, the composition change in the ileal microbiota became more dramatic over time.

Despite the importance of postbiotics in the regulation of various physiological and biochemical reactions in humans and animals have been well-known to date (34, 35), we barely understand the detailed and dynamic changes in postbiotics in pigs, such as change patterns of SCFAs and bioamines. This study tracked a significant gradual increase in only the total SCFA level in the gut but not in the total bioamine level during the early life of the pigs. The compositions of SCFAs and bioamines also varied during this period, such as the decreasing level of acetic acid and increasing levels of butyric acid and propionic acid observed (*P* < 0.05). Certainly, the gut microbiota is the critical factor for metabolites production, and the precise relationship between the intestinal microbiota and metabolites should be explored in future studies.

Combining the previous reports and our findings in this study, it has been believed that many different external or internal factors have a huge impact on the early intestinal development and gut microbiota establishment in pigs, including the live environment, immunization, drugs, nutrition, feed and even the physical form of the diet (3, 9, 20, 36). Colonization, succession, and function of the intestinal bacteria community in pigs, especially piglets, is directly or indirectly interfered by these factors. In this study, we have observed both creep feeding and weaning caused a significant changes in gut health and microbiota stable in the piglets at early life that would affect the weaned body weight and weaned survival rates of piglets. However, due to the complexity of the interactive process between gut microbiota and host, it is very difficult to distinguish the effects of all factors in the same study and that is a limitation in this present study.

On the basis of recognizing the composition and changes of intestinal microbiota in piglets, we are going to control and adjust the gut ecosystem reasonably. Probiotic, prebiotics, and the foods rich in dietary fiber or polyunsaturated fatty acids are applicable tools for the improvement and optimization of gut microbiota in animal husbandry (16, 37, 38). In addition, microbiota transplantation is also expected to be an effective intervention method for the re-shape of intestinal micro-ecology in animals (4, 39). Therefore, a systematic feeding strategy for the maintaining and improving of digestive function and intestinal health in piglets is necessary in the antibiotic-free feeding.

## Conclusions

During the early growth stage (1–42 days), the piglets grew quickly with gradual increases in blood levels of growth hormone and insulin, and in the intestinal developmental index and immunity. The alpha diversity of colonic microbiome community was higher than ileum. However, the composition change in the ileal microbiota was more dramatic over time. *Lactobacillus* genus was the dominant bacteria in piglets' ileum while *Prevotella* and *Ruminococcaceae* genera were the dominant bacteria in colon up to weaning. According to the changes in gut physiological index and intestinal microbiota in this study, the early growth of piglets can be divided into three phases: newborn, before wean, and post wean. In addition, the changes in gut microbiota significantly affected the amount and composition of bacterial functional metabolites such as SCFAs and bioamines. SCFAs showed greater changes in piglets at early life in comparison with bioamines. All these findings have important guiding significance for guarding pigs' health and increasing production performance.

## Data Availability Statement

All the original 16S rRNA sequencing data were submitted to the National Center for Biotechnology Information GenBank Sequence Read Archive database under accession number PRJNA640440.

## Ethics Statement

The animal study was reviewed and approved by the Ethics Committee of the Chongqing Academy of Animal Science (CAAS-S2018-11, Chongqing).

## Author Contributions

RQ and ZL designed this study. RQ, XQ, and LD carried out the animal experiments, sample collection, and detection. JW and JH were responsible for the 16sRNA sequencing, data analysis, and QW wrote the original manuscript. All authors contributed to the article and approved the submitted version.

## Conflict of Interest

The authors declare that the research was conducted in the absence of any commercial or financial relationships that could be construed as a potential conflict of interest.

## References

[1] Guevarra RB Lee JH Lee SH Seok MJ Kim DW Kang BN. Piglet gut microbial shifts early in life: causes and effects. J Anim Sci Biotechnol. (2019) 10:1. 10.1186/s40104-018-0308-3 30651985 PMC6330741

[2] Campbell JM Crenshaw JD Polo J. The biological stress of early weaned piglets. J Anim Sci Biotechnol. (2013) 4:19. 10.1186/2049-1891-4-19 23631414 PMC3651348

[3] Rodas B Youmans BP Danzeisen JL Tran H Johnson TJ. Microbiome profiling of commercial pigs from farrow to finish. J Anim Sci. (2018) 96:1778–94. 10.1093/jas/sky109 29635455 PMC6140882

[4] Hu L Geng S Li Y Cheng S Fu X Yue X. Exogenous fecal microbiota transplantation from local adult pigs to crossbred newborn piglets. Front Microbiol. (2017) 8:2663. 10.3389/fmicb.2017.02663 29375527 PMC5767267

[5] Le Sciellour M Renaudeau D Zemb O. Longitudinal analysis of the microbiota composition and enterotypes of pigs from post-weaning to finishing. Microorganisms. (2019) 7:622. 10.3390/microorganisms7120622 31795103 PMC6956163

[6] Yang H Xiao Y Wang J Xiang Y Gong Y Wen X. Core gut microbiota in Jinhua pigs and its correlation with strain, farm and weaning age. J Microbiol. (2018) 56:346–55. 10.1007/s12275-018-7486-8 29721832

[7] Wang X Tsai T Deng F Wei X Chai J Knapp J. Longitudinal investigation of the swine gut microbiome from birth to market reveals stage and growth performance associated bacteria. Microbiome. (2019) 7:109. 10.1186/s40168-019-0721-7 31362781 PMC6664762

[8] de Goffau MC Lager S Sovio U Gaccioli F Cook E Peacock SJ. Human placenta has no microbiome but can contain potential pathogens. Nature. (2019) 572:329–34. 10.1038/s41586-019-1451-5 31367035 PMC6697540

[9] Gresse R Durand CF Dunière L Blanquet-Diot S Forano E. 2019. Microbiota composition and functional profiling throughout the gastrointestinal tract of commercial weaning piglets. Microorganisms. (2019) 7:343. 10.3390/microorganisms7090343 31547478 PMC6780805

[10] Festi D Schiumerini R Eusebi LH Marasco G Taddia M Colecchia A. Gut microbiota and metabolic syndrome. World J Gastroenterol. (2014) 20:16079–94. 10.3748/wjg.v20.i43.16079 25473159 PMC4239493

[11] Gao J Xu K Liu H Liu G Bai M Peng C. Impact of the gut microbiota on intestinal immunity mediated by tryptophan metabolism. Front Cell Infect Microbiol. (2018) 8:13. 10.3389/fcimb.2018.00013 29468141 PMC5808205

[12] Lahiri S Kim H Garcia-Perez I Reza MM Martin KA Kundu P. The gut microbiota influences skeletal muscle mass and function in mice. Sci Transl Med. (2019) 11:eaan5662. 10.1126/scitranslmed.aan5662 31341063 PMC7501733

[13] Lobionda S Sittipo P Kwon HY Lee YK. The role of gut microbiota in intestinal inflammation with respect to diet and extrinsic stressors. Microorganisms. (2019) 7:271. 10.3390/microorganisms7080271 31430948 PMC6722800

[14] LeBlanc JG Chain F Martín R Bermúdez-Humarán LG Courau S Langella P. Beneficial effects on host energy metabolism of short-chain fatty acids and vitamins produced by commensal and probiotic bacteria. Microb Cell Fact. (2017) 16:79. 10.1186/s12934-017-0691-z 28482838 PMC5423028

[15] Sharon G Sampson TR Geschwind DH Mazmanian SK. The central nervous system and the gut microbiome. Cell. (2016) 167:915–32. 10.1016/j.cell.2016.10.027 27814521 PMC5127403

[16] Wegh CAM Geerlings SY Knol J. Postbiotics and their potential applications in early life nutrition and beyond. Int J Mol Sci. (2019) 20:4673. 10.3390/ijms20194673 31547172 PMC6801921

[17] Alldritt I Whitham-Agut B Sipin M Studholme J Trentacoste A Tripp JA. Metabolomics reveals diet-derived plant polyphenols accumulate in physiological bone. Sci Rep. (2019) 9:8047. 10.1038/s41598-019-44390-1 31142795 PMC6541599

[18] Xia B Shi XC Xie BC Zhu MD Chen Y Chu XY. Urolithin A exerts antiobesity effects through enhancing adipose tissue thermogenesis in mice. PLoS Biol. (2020) 18:e3000688. 10.1371/journal.pbio.3000688 32218572 PMC7141696

[19] Holman DB Brunelle BW Trachsel J Allen HK. Meta-analysis to define a core microbiota in the swine gut. mSystems. (2017) 2:e00004–17. 10.1128/mSystems.00004-17 28567446 PMC5443231

[20] Isaacson R Kim HB. The intestinal microbiome of the pig. Anim Health Res Rev. (2012) 13:100–9. 10.1017/S1466252312000084 22853934

[21] Liu H Zeng X Zhang G Hou C Li N Yu H. Maternal milk and fecal microbes guide the spatiotemporal development of mucosa-associated microbiota and barrier function in the porcine neonatal gut. BMC Biol. (2019) 17:106. 10.1186/s12915-019-0729-2 31852478 PMC6921401

[22] Choteau L Vancraeyneste H Le Roy D Dubuquoy L Romani L Jouault T. Role of TLR1, TLR2 and TLR6 in the modulation of intestinal inflammation and *Candida albicans* elimination. Gut Pathog. (2017) 9:9. 10.1186/s13099-017-0158-0 28289440 PMC5310049

[23] Gorvitovskaia A Holmes SP. Interpreting Prevotella and Bacteroides as biomarkers of diet and lifestyle. Microbiome. (2016) 4:15. 10.1186/s40168-016-0160-7 27068581 PMC4828855

[24] Kovatcheva-Datchary P Nilsson A Akrami R Lee YS De Vadder F Arora T. Dietary fiber-induced improvement in glucose metabolism is associated with increased abundance of Prevotella. Cell Metab. (2015) 22:971–82. 10.1016/j.cmet.2015.10.001 26552345

[25] Sudo N. Biogenic amines: signals between commensal microbiota and gut physiology. Front Endocrinol (Lausanne). (2019) 10:504. 10.3389/fendo.2019.00504 31417492 PMC6685489

[26] Koopmans SJ Schuurman T. Considerations on pig models for appetite, metabolic syndrome and obesetype 2 diabetes: from food intake to metabolic disease. Eur J Pharmacol. (2015) 759:231–39. 10.1016/j.ejphar.2015.03.044 25814261

[27] Roura E Koopmans S Lallès JP Huerou-Luron I de Jager D Schuurman T. Critical review evaluating the pig as a model for human nutritional physiology. Nutr Res Rev. (2016) 29:60–90. 10.1017/S0954422416000020 27176552

[28] Zegarra-Ruiz DF El Beidaq A Iñiguez AJ Lubrano Di Ricco M Manfredo Vieira S Ruff WE. 2019. A diet-sensitive commensal lactobacillus strain mediates tlr7-dependent systemic autoimmunity. Cell Host Microbe. (2019) 25:113–27. 10.1016/j.chom.2018.11.009 30581114 PMC6377154

[29] Zhao X Wang W Blaine A Kane ST Zijlstra RT Gänzle MG. Impact of probiotic *Lactobacillus* sp. on autochthonous lactobacilli in weaned piglets. J Appl Microbiol. (2019) 126:242–54. 10.1111/jam.14119 30276941

[30] Sun J Du L Li X Zhong H Ding Y Liu Z. Identification of the core bacteria in rectums of diarrheic and non-diarrheic piglets. Sci Rep. (2019) 9:18675. 10.1038/s41598-019-55328-y 31822779 PMC6904459

[31] Lu D Tiezzi F Schillebeeckx C McNulty NP Schwab C Shull C. Host contributes to longitudinal diversity of fecal microbiota in swine selected for lean growth. Microbiome. (2018) 6:4. 10.1186/s40168-017-0384-1 29301569 PMC5755158

[32] Yang H Huang X Fang S He M Zhao Y Wu Z. Unraveling the fecal microbiota and metagenomic functional capacity associated with feed efficiency in pigs. Front Microbiol. (2017) 8:1555. 10.3389/fmicb.2017.01555 28861066 PMC5559535

[33] Zhao W Wang Y Liu S Huang J Zhai Z He C. The dynamic distribution of porcine microbiota across different ages and gastrointestinal tract segments. PLoS One. (2015) 10:e0117441. 10.1371/journal.pone.0117441 25688558 PMC4331431

[34] Fluitman KS Wijdeveld M Nieuwdorp M IJzerman RJ. Potential of butyrate to influence food intake in mice and men. Gut. (2018) 67:1203–4. 10.1136/gutjnl-2017-315543 29382775 PMC6031269

[35] Hartstra AV Nieuwdorp M Herrema H Interplay between gut microbiota, its metabolites and human metabolism: dissecting cause from consequence. Trends Food Sci Technol. (2016) 57:233e243 10.1016/j.tifs.2016.08.009

[36] Cappai MG Dimauro C Arlinghaus M Sander SJ Pinna W Kamphues J. Subluminal focal lesions in peyer's patches in the terminal ileum of pigs fed with different physical forms of one same diet. Front Vet Sci. (2020) 7:207. 10.3389/fvets.2020.00207 32478102 PMC7242563

[37] Cappai MG Wolf P Pinna W Rust P Kamphues J Pre-caecal disappearance of starch and volatile fatty acid (VFA) content in digesta of caecum of growing pigs fed with ripe hulled shredded acorns in their diet. Agriculture (Switzerland). (2020) 11:508 10.3390/agriculture10110508

[38] Sanders ME Merenstein DJ Reid G Gibson GR Rastall RA Probiotics and prebiotics in intestinal health and disease: from biology to the clinic. Nat Rev Gastroenterol Hepatol. (2019) 16:605–16. 10.1038/s41575-019-0173-3 31296969

[39] Wargo JA. Modulating gut microbes. Science. (2020) 369:1302–3. 10.1126/science.abc3965 32913089

